# Naturalistic Parent Teaching in the Home Environment During Early Childhood

**DOI:** 10.3389/fpsyg.2022.810400

**Published:** 2022-03-21

**Authors:** Sandra L. Della Porta, Putri Sukmantari, Nina Howe, Fadwa Farhat, Hildy S. Ross

**Affiliations:** ^1^Department of Educational Studies, Brock University, St. Catharines, ON, Canada; ^2^Department of Education, Concordia University, Montreal, QC, Canada; ^3^Department of Psychology, University of Waterloo, Waterloo, ON, Canada

**Keywords:** parent–child teaching, early childhood, home environment, domains of learning, sociocultural theory

## Abstract

Children’s sociocultural experiences in their day-to-day lives markedly play a key role in learning about the world. This study investigated parent–child teaching during early childhood as it naturally occurs in the home setting. Thirty-nine families’ naturalistic interactions in the home setting were observed; 1033 teaching sequences were identified based on detailed transcriptions of verbal and non-verbal behavior. Within these sequences, three domains of learning (knowledge, skills, and dispositions) and subtopics were identified and analyzed in relation to gender, child birth order, context, teaching strategies, and learner response. Findings show knowledge, skills, and dispositions were taught equally, marked by the most prominent subtopics taught within each domain, including cognitive (skill), game rule (knowledge), and social rule (disposition). Further, mothers and fathers were found to teach their children equally, however, fathers taught knowledge more than mothers, whereas mothers taught dispositions more than fathers. Differences between domains of learning and subtopics also existed between mother’s and father’s teaching based on child birth order and gender. This study also assessed the contrast between teaching knowledge, skills, and dispositions by context, parent teaching strategies, and child learner response. Results support the notion that family interactions in the home setting set a stage for children’s rich informal learning experiences. Vygotskian sociocultural conceptions underpin this research and findings are discussed using this central theoretical lens.

## Introduction

Children are exposed to a wealth of learning experiences through interactions with significant others (Hartup, 1989), and as they spend most of their early years with their family, this close-knit context has a significant impact on their development. Thus, parents serve a vital role in children’s development and significantly contribute to their children’s knowledge and attitudes about the world (Bornstein, 2015). At the crux of parents’ socialization of young children is the process of transferring knowledge, teaching skills, and facilitating socially and morally sound comportment. Ziv et al. (2016) theorize teaching is an intentional effort to influence learner’s knowledge and beliefs, thus when parents teach their children, there is an expected change in the learner’s understanding and/or behavior. Studies have investigated parent-teaching in semi-structured settings or by parental scaffolding of specific skills (Bell et al., 1981; Pellegrini et al., 1985; LeBlanc and Bearison, 2004; Bornstein, 2015). However, naturalistic interactions at home go largely undocumented (for an exception see Farhat et al., 2021), particularly regarding *what* parents teach and *how* parents approach teaching. Our novel study investigated parental teaching during naturalistic ongoing interactions at home, focusing on three domains of learning: knowledge, skills, and dispositions, and related subtopics. In addition, we aimed to identify differences in maternal and paternal teaching of said topics and differences based on children’s birth order and gender. Differences in context, teaching strategy, and learner responses connected to domains of learning were also examined. Data were collected in the early 1990s, providing a window into the teaching and learning process between parents and children at the time, informing our present knowledge in the area.

### Sociocultural Perspective

Sociocultural theorists posit children learn through active involvement and social interactions with others in relevant situations (Vygotsky, 1978; Rogoff, 1990; Bligh and Fathima, 2017; Vandermaas-Peeler et al., 2019). From this perspective, children’s learning is related to their social world and a connection between the social and the individual (Vygotsky, 1978; Rogoff, 1990). This interaction is a medium in which children can learn and acquire new skills within their zone of proximal development (ZPD), through guided participation from a more experienced partner (Vygotsky, 1978; Rogoff, 1990). These more knowledgeable others are an integral part of children’s learning (Vygotsky, 1978), as they intentionally engage in an activity to increase the knowledge of the less experienced partner. Strauss and Ziv (2012) define teaching as a form of social learning focused on cognition and development, with theory of mind at the center of this process.

This study adds to the literature by identifying *what* parents are teaching their children and *how* the teaching process varies based on socially constructed behavior (e.g., gender norms and expectations) and situational context (e.g., actors involved) in the home setting. In line with this theory, we argue that an essential way in which we understand children is through the transfer of knowledge from their sociocultural worlds, which in early childhood begins with parents’ teachings in a non-digitized setting. Vygotsky emphasized, “the dominant role of social experience in human development” (Vygotsky, 1978, p. 22), supporting the notion that children’s learning is naturally embedded in their close social relationship interactions. It is about what happens *between* minds (i.e., parent and child), the higher mental functions being stimulated and developed, that transfers social and cultural meaning to children (Fuhrer and Josephs, 1998). Through these joint cognitive processes with parents, as sociocultural experts, children internalize meaning and transfer knowledge to other contexts (Fuhrer and Josephs, 1998). This paper adds to our understanding of children’s development as it is fostered through sociocultural means.

### Parent Teaching

Underscoring Vygotsky’s sociocultural theory, Bornstein (2015) emphasizes parents’ role as children’s first teachers to help build their understanding of the social, emotional, cognitive, and physical world. Parenting involves teaching children about their surroundings, introducing and facilitating an understanding, demonstrating, and offering opportunities to explore and imitate, all with the collective goal of health, achievement, and social and economic well-being (Bornstein, 2015). Through guided participation and by providing opportunity to engage in select activities, parents set the stage for children to socially construct understanding of cultural tools in their society (Rogoff, 1990; Stone, 2004). In this sense, parents play a significant role as the more experienced partners to ensure children’s learning within their ZPD during daily natural interactions and experiences (Hedges, 2021). The countless social interactions within the home afford opportunities for natural, authentic, and situated learning. Parent–child interactions provide opportunities for relevant and informal situated learning to emerge. As Lave (1991) suggested “conditions for learning flourish in the interstices of family life” (p. 78).

### Topics Taught in the Home Setting

We presently have a narrow empirical view as to the breadth of what children are learning in the home setting. This paper utilized Katz’s (1986) three proposed goals of education: knowledge, skills, and dispositions, to identify and organize learning translated to children through parent–child teaching. Similarly, Bloom et al. (1956) categorized three domains of learning: cognitive (knowledge), psychomotor (skills), and affective (attitude). Knowledge as a cognitive domain is explained as a thinking process (Hoque, 2016) or “content of mind” (Katz, 1986, p. 5). Knowledge can be procedural (e.g., how to play a game, operate a toy) or conceptual (what) in relation to understanding the world (Hatano and Inagaki, 1986; Howe et al., 2015). Skills are behaviors that can be acquired through practice or learned strategies to function in the world, such as writing, arithmetic, or mental skill (Katz, 1986). Though learning can happen everywhere (e.g., family, community, organization), school tends to take responsibility for the acquisition of academic knowledge and skills, while dispositions are not usually listed in curriculum goals (Katz, 1993). Dispositions refer to mindset, habits of minds, or comportment (Katz, 1986, 1993). While skills can be learned via drill and practice, dispositions are learned through observation (i.e., modeling) and punishment or reinforcement (Katz, 1986). A disposition (e.g., positive regard for others) must be enacted for it to be nourished, therefore, to encourage healthy dispositions, opportunities to enact them must be offered. Hoque (2016) explains this in the affective domain of learning, which deals with emotions, values, motivation, or attitudes. Though knowledge and skills are commonly known to be the goal of teaching, Katz (1993) argued for the importance of dispositions for young children as knowledge and skills alone are not sufficient to ensure they are being applied in life and interactions with others.

### Parent Teaching of Knowledge, Skills, and Dispositions

Though the majority of parent-teaching literature focuses on knowledge and skills, particularly academically related, several scholars identify the positive aspects and need to investigate dispositional teaching. Studies have examined parental-teaching of knowledge and skills regarding numeracy (Vandermaas-Peeler et al., 2009, 2012), literacy (Sénéchal et al., 1998; Sénéchal and LeFevre, 2002; Hood et al., 2008) or both (Skwarchuk et al., 2014). The focus of these studies may be due to the growing body of empirical findings indicating that early understanding of literacy and numeracy predict academic success (Duncan et al., 2007; Tamis-LeMonda et al., 2019). Furthermore, there is a positive relationship between the quality of the home environment and children’s social (dispositions) and academic (knowledge and skills) outcomes (Organisation for Economic Co-operation and Development [OECD], 2010; Skwarchuk et al., 2014).

Other research indicates parents teach safety (disposition) and how to differentiate safe and dangerous situations (Morrongiello et al., 2014). Bornstein (2015) proposes parents concentrate on their children’s physical (motor skills) development and social (disposition) development, including emotion regulation, feeling valued, style of communication, and interpersonal ability to engage in a healthy relationship. With concern for children’s well-being and welfare (disposition), scholars suggest parents highlight the teaching of self-regulation, self-esteem, empathy, self-control, and treatment of others, which fall under the moral domain and prosocial behavior (Smetana, 1999; Lavoie et al., 2016). Moreover, descriptive research found parents’ early socialization practices involved fostering children’s dispositional behavior (e.g., household responsibility, prosocial behavior, and aggression) and cognition, specifically regarding language and motor skills (Power and Parke, 1986). Smetana (1999) indicates that parents are focused on developing morally and socially accepted behavior (disposition) in their children. Lastly, research shows a maternal tendency to direct teaching to ensure the safety of the child, before focusing on socially acceptable behavior (disposition) (Gralinski and Kopp, 1993). Clearly, teaching these attitudes, habits of mind, and comportment are an important area of learning. Whether and how parents teach children about these domains during ongoing naturalistic interaction at home is not known.

### Parent–Child Teaching and Family Dynamics: Gender and Birth Order

Parent–child teaching is closely related to the dynamics and composition of the family: specifically, parents’ and children’s gender and sibling birth order (Maccoby and Jacklin, 1974; Block, 1984; McGillicuddy-De Lisi, 1988). Researchers have studied approaches of maternal and paternal teaching and documented mothers to be more alert to children’s cues and to use more reasoning strategies with their child than fathers, whereas fathers employed more direct statements to control behavior (McLaughlin et al., 1980; Power, 1985). McLaughlin et al. (1980) examined parental speech during a game and reported that fathers used more direct, controlling types of language (imperatives, direct suggestions, and prompting questions) than mothers (indirect suggestions, information questions, and rule clarifications). This study also revealed fathers directed more authoritarian orders or commands to sons than daughters. Paquette and Bigras (2010) investigated parental behavior in risky situations faced by children (from social risk to physical risk such as climbing stairs) and found mothers to be more involved in disciplining children and doing arms-length supervision compared to fathers; both parents tended to respond to the demands of daughters more than sons. Further, during play, fathers tended to support their children’s risk taking, whereas mothers taught perspective taking (Clarke-Stewart, 1978; Power et al., 1994).

Others have investigated parents’ approach to teaching according to child gender. Block (1984) reported parents’ use of high-level distancing strategies differed between sons and daughters, placing the male child in control by encouraging planning, decision making, and independent thinking. During a cognitive task constructing squares, parents used this strategy more with sons and demanded more achievement from sons than daughters (Block). This provides boys with the opportunity to engage with the task with cognitive independence, whereas daughters were provided instruction, without cognitive challenge. Relatedly, McGillicuddy-De Lisi (1988) documented based on a paper folding task that parents demanded high-level cognitive skills (inferencing, generalizing, finding alternative) from children of the opposite sex from that of the parents. This finding is contrary to Maccoby and Jacklin’s (1974) study of parental demands during teaching highlighting the “same-sex severity, opposite-sex indulgence rule” (p. 158); namely, fathers are more demanding of sons and more permissive of daughters (also see Bell et al., 1981). In contrast, Maccoby and Jacklin noted mothers impose higher demands on their daughters than sons. Nevertheless, Pellegrini et al.’s (1985) study showed no differences in mothers’ and fathers’ language or teaching strategy based on children’s gender.

Furthermore, early socialization research by Power and Parke (1986) indicated that in a naturalistic home setting both parents teach prosocial behavior to daughters and household rules to sons; mothers tend to teach household rules more than fathers. A review of the literature by Paquette (2004) indicated fathers’ presence, active engagement, and rough and tumble play improves children’s positive risk-taking behavior, life skills, and controls aggression while mothers tend to solve problems for the child without providing opportunities for them to learn the skill. Though these findings are not focused on explicit teaching, they provide implicit indications of indirect teaching through engagement and play. Other topics comparing mothers and fathers’ similarities and differences in teaching their children have yet to be investigated.

Limited research also examines parental teaching behavior directed toward older and younger children. According to Pleck (1997), fathers and mothers tend to be less involved and spend less time with older than younger children. Conversely, Monteiro et al. (2017) found fathers are more involved in play with first-born than second-born children. These authors reported a difference in parental responsive scaffolding based on their child’s competency; parents took more control over conversations in which the child was less competent, whereas parents were less demanding, used fewer cognitive strategies, asked more questions, and were more directive with more competent children (Pellegrini et al., 1985). This finding may inform parents’ tendency to spend more time and attention with younger children who, at least in comparison to an older sibling, may need more teaching and guidance.

### Parent Teaching Context, Strategies, and Learner Response

In terms of context, McBride and Mills (1993) found consistency in mothers’ focus on child rearing and time spent in functional work-related interaction, whereas fathers interacted in playful activities. Further, mothers teach their children more during contingent activities such as caregiving and leisure and fathers teach skills and play games (Pleck, 1997; Monteiro et al., 2017). Farhat et al. (2021) recently investigated parent–child teaching during naturalistic interactions at home and reported differences in mothers’ and fathers’ teaching in different contexts; mothers taught more during conflict, fathers during games.

Farhat et al. (2021) identified the following teaching strategies in parent–child teaching: direct instruction, labeling, demonstration, suggestion, explanation, positive feedback, negative feedback, and questioning. Parents opted for direct instruction and labeling but there were no differences between mothers’ and fathers’ employment of teaching strategies. From the perspective of children as learners, Farhat et al. (2021) found learners’ responses were either characterized by active involvement or no response; these two responses occurred significantly more than children’s responses of compliance or rejecting the teaching.

Rothbart and Rothbart (1976) conducted a study of mothers’ supervision during memory and puzzle tasks and found mothers’ encouragement differed according to the children’s gender. Mothers ignored sons’ request for help and responded more to mistakes made by girls and attended to their help-seeking. This finding suggests mothers helped girls more than boys in their problem-solving tasks. Block (1983) summarized a comparison of parents of boys and of girls from several studies and determined that fathers emphasized interpersonal relationships with daughters and were more controlling with sons. Another study suggests both parents stressed achievement more for sons than daughters (Block, 1984).

Research on parent–child teaching generally uses semi-structured paradigms where parents engage in a certain activity to teach a specific skill or to investigate parental scaffolding (Rothbart and Rothbart, 1976; Bell et al., 1981; Pellegrini et al., 1985; LeBlanc and Bearison, 2004; Bornstein, 2015). Though research on naturalistic parent–child teaching during ongoing interactions is limited, there is evidence of the ways parents teach their children. Moreover, it is not empirically known *what* topics parents teach children during naturalistic ongoing interactions. This knowledge informs our understanding of meaning translated and cultural tools fostered in children’s central context to early learning, namely family interactions in the home setting.

### The Present Study

The present study concentrates on the sociocultural perspective (Vygotsky, 1978; Rogoff, 1990) as the framework to investigate naturalistic parent–child teaching using Katz’s (1986) three goals of education: knowledge, skills, and dispositions. We compare domains of learning and subtopics taught by mothers and fathers, within which context teaching occurred, what teaching strategies were most likely to be used in teaching a specific topic, and how learners responded. Gender composition and birth order were also investigated to determine whether parents’ approach and strategies differed when teaching their children.

Six inquiries guided this study. First, we sought to identify common domains of learning (i.e., knowledge, skills, and dispositions). Following the growing body of study in literacy (e.g., Sénéchal and LeFevre, 2002; Hood et al., 2008), numeracy (e.g., Vandermaas-Peeler et al., 2009, 2012), and Katz’s (1993) framework, we hypothesized parents would be concerned with their children’s school readiness and focus more on teaching knowledge and skills than dispositions. Second, to detect differences between mothers’ and fathers’ teaching of the domains of learning, we predicted mothers would teach dispositions more than fathers based on literature revealing mothers are more involved in disciplining and enforcing household rules (Power and Parke, 1986; Paquette and Bigras, 2010). Pleck (1997) also indicated fathers spent more time teaching skills and playing games, while Paquette (2004) indicated fathers’ tendency to reinforce their children in trying a new skill. In this vein, we speculated fathers would teach knowledge and skills more than mothers. Third, to address how fathers or mothers approach teaching based on child birth order, we speculated parents would teach younger more than older siblings, following a Vygotskian approach according to competency (Pellegrini et al., 1985). In relation to gender, we predicted fathers would teach skills more with sons (Block, 1983), whereas mothers would teach skills more with daughters, based on the same-sex severity, opposite-sex indulgence notion (Maccoby and Jacklin, 1974). Fourth, to establish the context of teaching certain domains of learning, we predicted based on Farhat et al. (2021), knowledge and skills would be taught more often during games and dispositional behavior during conflict or contingent activity. Fifth, as Katz (1986) argued, children strengthen skills through practice and dispositions through reinforcement. Thus, we speculated parents would teach skills using demonstration, whereas to teach dispositions, parents would use direct instruction, positive, and negative feedback. Sixth, we aimed to determine whether children’s responses to teaching differed based on topic taught. To our knowledge, no studies have investigated *differences* between knowledge, skills, and dispositions taught in naturalistic settings, but several studies (Howe et al., 2015, 2016; Farhat et al., 2021) demonstrated children respond most frequently by being actively involved or not respond during teaching. Thus, we could not predicted differences in responses by topics taught, but do predict overall active involvement and no response would be the most common responses in the three domains of learning.

## Materials and Methods

### Participants

Families were recruited from a midsized urban city in Ontario, Canada, through birth announcements in the local newspaper, contacting preschools, and passive snowball sampling. The study was powered to detect large-sized effects (*f* > 0.60) with 39 participating families. To be eligible to participate, families were required to have two children approximately 4 and 6 years old. Families were predominantly middle-class, Caucasian with two parents and at least two children under age seven. Older siblings’ age ranged from 5.4 to 7.0 years (*M* = 6.3 years, *SD* = 0.42), while the younger siblings’ age ranged from 3.8 to 4.7 years (*M* = 4.4, *SD* = 0.21). The age gap between siblings was *M* = 1.94 years, *SD* = 0.28. Sibling gender (as identified by parents) was as follows: older (male = 19, female = 18), younger (male = 18, female = 19). Mother’s *M*age = 32.8 years, father’s *M*age = 34.6 years. Parents’ education ranged from university (29%), community college (15%), high school (41%), to no high school diploma (15%). Although data collection focused on natural family interaction, fathers were absent in half of the sessions. In the present study, sessions were included that involved both parents (*n* = 111), while sessions without fathers’ presence were excluded (*n* = 132). Ethical approval for the original data collection, which began in 1989, was obtained from the University of Waterloo (see Ross et al., 1994); secondary data analysis was granted approval from Concordia University.

### Procedure

#### Observational Data Collection

The original data collection procedure entailed observations of families’ interaction in their homes for a total of 9 h over six sessions. Two trained observers recorded each family’s interactions for 20-min preliminary sessions preceding the actual data collection to familiarize the family with the observation process; ten of these sessions were used to determine reliability. During the observations, each family was assigned one observer who used a two-track audio recorder; an omnidirectional microphone recorded the family’s verbal communications, and the other microphone recorded the observer’s descriptions of the participants’ behavior. The observer was trained to be discreet, both parents and children were asked to ignore the observer. Families were asked to avoid distractions such as guests or electronic devices (e.g., television, video games). Sessions were transcribed after each visit, identifying actors, targets, and action moves. In the transcription process, 96 verbal and non-verbal behaviors, interaction contexts, and speech were coded (see Ross et al., 1994).

#### Identification of Teaching Sequences

Researchers utilized sessions involving both mothers and fathers to identify parent teaching sequences, which were identified when parents intentionally taught the child or children, either explicitly or implicitly (see Table 1 for sample teaching sequence). These sequences appeared as direct teaching (“Seven’s higher than six”), indirect teaching (“When I twirl you, how do I do it?”), corrective teaching (“Move the other way”), and a reprimand with explanation (“Bucking horses hurt people!”). Sequences were not coded when parents engaged in an action without a verbal explanation (tying shoes without speaking), basic conversations occurred without a clear intent to teach, the scenario was ambiguous, parents asked for information (“What is his name?”) with no follow-up teaching, or during pretense.

**TABLE 1:** Sample teaching sequence.

Actor	Target	Behavior code	Action	Teaching strategy code	Domain of learning code
F	Y	Ask Information	You’re still past the line aren’t ya?	Suggestion/Clarification	
Y	F	Disagree Verbally	No		
F	Y	Ask Information	James, where’s the line?	Suggestion/Clarification	
Y	F	Suggested Action	How about I make the line right here?		
Y	F	Show Non-verbally	(points to the spot closer to the target marble)		
F	Y	Protest Verbally	James, you can’t.	Negative Feedback	
F	Y	Invoke Rule	That’s where we’re all shooting from, behind the line.	Labeling	Knowledge: Game Rule
F	Y	Ask Information	Now, do you want to play?	Suggestion/Clarification	
Y	F	Disagree Verbally	No, it’s not…		
F	Y	Threat	Okay, we have to disqualify you, we’re gonna have to not play anymore.	Negative Feedback	
F	Y	Justify	Cause we can’t play with somebody that’s not gonna play fair.	Explanation	Disposition: Prosocial
Y	F	No Response			
F	Y	Ask Information	What do you think, Mike?	Suggestion/Clarification	Skill: Cognitive
Y	F	No Response			
F	Y	Ask Information	Are you gonna play fair?	Suggestion/Clarification	
Y	O	Positive Other	What did you say the last time?		
O	Y	Positive Other	I don’t know		
Y	O	Describe Object	You know		
F	Y	Describe Action Other	He said you shouldn’t go past the line	Labeling	
F	Y	Insult	He doesn’t want to play with somebody that doesn’t play fair	Explanation	

*Father (F), older (O) and younger (Y) sibling are playing a marble game. Participant names were changed to pseudonyms. Coding was as follows: Teacher = F; Learner = Y; Domain of Learning (Subtopics): Knowledge (Game rule), Skill (Cognitive), Disposition (Prosocial) (codes are placed in the table in areas where the domain is clearest, though indications of learning domains do occur throughout the sequence); Context: Conflict; Teaching Strategies: Labeling, suggestion/clarification, explanation, negative feedback; Learner Response = Active Involvement.*

Initiation of the sequences commenced either by parent(s) or child(ren). Parents could spontaneously teach, by correcting or reminding the child(ren), or intentionally sharing information with the child(ren). Children’s responses were identified keeping in mind an attempt to teach might not have elicited an explicit response. Context was considered in identifying the start and end line of each sequence. If the topic changed, this signaled the end of the sequence, and a new sequence was identified. In some cases, the sequence ended when the child(ren) stopped responding or agreed to the teaching, parents checked whether the child(ren) comprehended, or parents praised the child(ren). Interrater reliability on 20% of the observed sessions was obtained by aligning the identified sequence turns by the two coders, Cohen’s *K* = 0.72, *p* < 0.001. For example, if RA A identified a sequence between lines 233–256, whereas RA B identified lines 232–257, 21 conversational turns were agreed upon and two were counted as a disagreement. Following the sequence identification process, 1033 teaching sequences showed evidence of teaching, after which each sequence was categorized into the three domains of learning (knowledge, skills, and dispositions) and 11 respective subtopics.

### Behavioral Coding

#### Actor Roles and Context

Actor and target (father, mother, older child, younger child), as well as context (i.e., game, contingent activity, pretense, and conflict) (see Table 2), were coded in the original transcription (Ross et al., 1994). Reliability for ten 20-min preliminary sessions showed a percentage agreement of actor and target (88%), behaviors (86%), and context (95%).

**TABLE 2:** Definitions and examples of contexts, teaching strategies, and child’s responses.

		Definition	Examples
Context	Contingent Activity	An action following another action.	Teaching how to eat or sit properly during a meal.
	Conflict	Incompatible behavior between two partners where an action of one is met with protest, resistance, or retaliation by another.	A parent teaching during a conflict between two children.
	Game	Playful interactions where all participants take on active roles.	Teaching how to hold cards during card game, game rule in a board game, or not to cheat during a marble game.
Teaching Strategies	Direct Instruction	Verbal or physical instruction including a response of yes or no.	F: “Put it on the floor then open the door”
	Labeling	Naming an object or an action/behavior	M: “Looks like a hard hat” M: “It’s wood”
	Demonstration	Showing how to do something in verbal or non-verbal way	Demonstrating how to hold the cards so others cannot see
	Positive Feedback	Praise or agreement	M: “Good for you, Beth” F: “That’s a good idea”
	Negative Feedback	Correction or negative feedback	M: “Not down the tube”
	Metacognitive Strategies	Using strategies that require learner’s thinking such as questioning, providing suggestions, or hinting a potential solution.	M: “Okay Meagan, they’re supposed to go into the back of his bowl in his truck” Y says does not know where to start, M “Why don’t you start with the blocks?”
Learner Response	Active Involvement	Learner actively asks questions, comments, extends, and builds on parent’s idea	F corrects a child’s direction in a board game and the child responds by moving in the direction and count the steps.
	Compliance	Agreeing with teaching without making further extension	The child says “Yes” or does what is told.
	No Response	Not giving responses or ignoring teaching	M reminds the child not to give ultimatums to F, the child ignores by talking to sibling about balloons.

#### Domains of Learning and Subtopics

Researchers met regularly to review teaching sequences and gain a mutual understanding of the definitions of knowledge, skills, and dispositions based on Bloom et al. (1956) and Katz’s (1986) domains of learning. The first phase involved identifying whether a teaching sequence involved knowledge, skills, and/or dispositions. Interrater reliability was calculated on 211/1033 (20%) of teaching sequences; Cohen’s *Kappas* were: knowledge (0.75), skills (0.75), and dispositions (0.84). The second phase involved identifying a subtopic. This step resulted in an exhaustive list of 22 subtopics branching from the three domains of learning. In the coding process, researchers would return to previously identified subtopics for refinement. Examples of teaching sequences from each subtopic were listed and added during the process. Once all sequences were categorized and reviewed, the researchers came to an agreement to merge subtopics based on shared attributes, narrowing the list to 11 subtopics (see Table 3): knowledge (conceptual knowledge, early academic concepts, game rules, and explaining a problem), skills (cognitive skills, problem solving and strategizing, early academic skills, and fine and gross motor skills) and dispositions (prosocial, social and household rules, and self-control).

**TABLE 3:** Definitions and examples of subcategories of parental teaching for each domain of learning.

		Definition	Examples
Knowledge	Conceptual knowledge	General concept about the world	F: “Dinosaurs used to live here before we did. Millions years ago the earth is home to dinosaurs. They were the biggest animals ever existed.”
	Early academic concept	Concept related to understanding of math	F: Shows what a half scoop is by lifting dough and explains that one quarter is half of a half
		Concept related to understanding of literacy	Y: “What is an emergency?” O: “I think that means…” F: “Special. It means special” M: “Something happens that they need you”
	Game rules	Explanation of game rules how to play	F: Explains because O won, he goes first, then order of players is clockwise.
	Explaining a problem	Explanation how a problem occurs without offering strategy to solve it	M: “That’s what happens when you put too much milk on it”
Skills	Cognitive skills	Opportunity for learners to think more in-depth and critically	F: “If you made a big tower, would she knock it over?”
	Problem solving and strategizing	Recognizing a problem and finding solutions	M: “Why don’t you move the broom, so no one lands on it and break it”
	Early academic skills	Ability to do math (e.g., counting)	Y: Rolls and then jumps his game piece around the board, F shows him how to count
		Ability to do literacy (e.g., spelling)	F: “How do you spell ‘NO’?”; F: “How do you spell ‘YES’?”
	Fine and gross motor skills	Ability to do a physical activity using fine and gross motor skills	F: Shows OY how to shuffle the cards by splitting the decking in half and spreading them out with the thumb
Dispositions	Prosocial and moral behavior	Behavior that is deemed right, good, or positive	F: “If you can’t play properly, don’t play”
	Social and household rules	Following rules set at home and in social-cultural context	M: “Are you gonna help clean this mess? I guess you don’t want to play with the puzzle anymore. If she doesn’t help, she doesn’t get to play”
	Self-control	How to control and regulate oneself to fit in the social world	M: “Daniel, instead of calling mommy for things like that, I think you should go work it out by yourself, okay?”

A single teaching sequence could be coded under one or multiple topics or subtopics. Of the 1033 teaching sequences, 929 were listed under a single subtopic, 96 under two subtopics (e.g., game rules with prosocial; cognitive skills with prosocial; cognitive skills with social and household rules), and 8 under three subtopics (e.g., game rules, prosocial, and early academic concept; cognitive skills, social and household rules, and self-control).

#### Teaching Strategies

Based on Farhat et al.’s (2021) coding scheme, eight parental teaching strategies were coded: (a) direct instruction (“You have to put another leg in the corner”), (b) labeling (“That’s the bull”), (c) suggestion/clarification (“Why don’t you start with the blocks?”), (d) verbal or non-verbal demonstrations (showing how to shuffle cards), (e) explanations (“Put it on top so it won’t fall over”), (f) questioning (“When I twirl you, how do I do it?”), (g) positive feedback (“That’s right”), and (h) negative feedback (“That’s not very nice.”) (see Table 2). Parents could employ more than one strategy while teaching, therefore in some cases, multiple strategies emerged in one sequence. Reliability was calculated with *Kappa* by Farhat et al. (2021): direct instruction = 0.68; labeling = 0.77; suggestion = 0.73; demonstrations = 0.73; explanations = 0.71; questioning = 0.91; positive feedback = 0.79; and negative feedback = 0.73.

#### Children’s Responses to Teaching

The children displayed various responses to parental teaching. Farhat et al. (2021) described a gradation of responses based on a child’s level of involvement. The highest level was active involvement, followed by compliance to teaching, explicit rejection to what was taught, and the lowest level was no response. *Kappa* for older response was 0.80; younger response was 0.76. In the event of several types of responses emerging in one sequence, the highest level of involvement was coded (e.g., an initial rejection that ended with active involvement would be coded as active involvement) (see Table 2).

## Results

Teaching sequences (*N* = 1033) were evident in 37/39 families. Sessions involving teaching sequences ranged from 1 to 5 (*M* = 2.70) per family, comprising between 4–110 sequences per session. Data were calculated using proportion scores with the family as the unit of analysis. For example, to compare domains of learning, the number of sequences of teaching knowledge was divided by the total number of teaching sequences (i.e., knowledge + skills + dispositions). The same calculation was employed for skills and dispositions. Repeated measures (RM) ANOVAs were conducted for all analyses, except for those in which a different testing procedure is identified. Bonferroni correction was utilized for RM ANOVAs and degrees of freedom were corrected using Greenhouse-Geisser estimates, where necessary. Significant results with a *p* value of between 0.05 and 0.10 were reported as trends.

Due to low frequency (<5%), the following codes were removed from analyses: learner response of rejection (*M* = 0.05, *SD* = 0.08) and pretense context (*M* = 0.03, *SD* = 0.06). Sequences in which mothers and fathers co-taught (*N* = 64) (6% of total sequences) qualitatively differed compared to independent parent–child teaching as parents likely influence each other by their mere presence. *Post-hoc* coding of co-teaching dynamics indicated: mothers and fathers repeated each other’s teaching (*n* = 22), fathers dominated (*n* = 10), mothers dominated (*n* = 9), fathers expanded on mother’s initial teaching (*n* = 7), and mothers expanded on father’s initial teaching (*n* = 16). Therefore, instances where parents co-taught were included for within factor analyses, however, were not included in comparative analyses between factors.

### Domains of Learning and Subtopics

Counter to our expectation that parents would teach knowledge and skills more than dispositions, no significant difference was found between knowledge (*M* = 0.38, *SE* = 0.04), skills (*M* = 0.38, *SE* = 0.03), and dispositions (*M* = 0.33, *SE* = 0.03).

Filling the gap in the empirical literature (i.e., no hypothesis posited), a comparison between subtopics taught indicated cognitive skills (*M* = 0.23, *SE* = 0.03) were taught most often, followed by game rule knowledge (*M* = 0.22, *SE* = 0.04), social and household rules (*M* = 0.20 *SE* = 0.03), *F*(3.29, 118.35) = 14.84, *p* < 0.001, $\eta_{\text{p}}^{2}$ = 0.29. These three subtopics were taught significantly more than all other subtopics, including prosocial disposition (*M* = 0.10, *SE* = 0.02), conceptual knowledge (*M* = 0.09 *SE* = 0.01), academic skills (*M* = 0.07, *SE* = 0.02), problem solving skills (*M* = 0.07 *SE* = 0.02), academic concepts (*M* = 0.05, *SE* = 0.01), self-control (*M* = 0.04, *SE* = 0.01), explanation of a problem (*M* = 0.03 *SE* = 0.01), and fine and gross motor skills (*M* = 0.02, *SE* = 0.01).

Of the total teaching sequences (*N* = 1033), 96 sequences involved two subtopics and eight sequences included three subtopics. No pattern of co-taught topics was evident across sequences. Displaying the intertwining nature of topics taught in the teaching/learning process, the most common combination of topics taught was game rules and prosocial behavior (*n* = 12) (e.g., parent reiterates the rules to a game *and* indicates it is not right to cheat) and cognitive skills and prosocial behaviors (*n* = 12) (e.g., parent states that it is not nice to threaten someone *and* asks child to consider how it would feel if someone threatened them), followed by cognitive skills and social or household rules (*n* = 10) (e.g., parent states household rule for sharing *and* explains why that situation requires consideration of others’ perspectives), game rules and early academic skills (*n* = 8) (e.g., parent indicates the game rule *and* checks child’s understanding of number order and value to best learn the rule), and game rules and cognitive skills (*n* = 7) (e.g., parent reminds child of the ‘no slap-shots’ rule in the basement *and* provides reasoning for the rule). All other categories co-occurred in four or fewer instances.

### Parent Teaching

**FIGURE 1:**
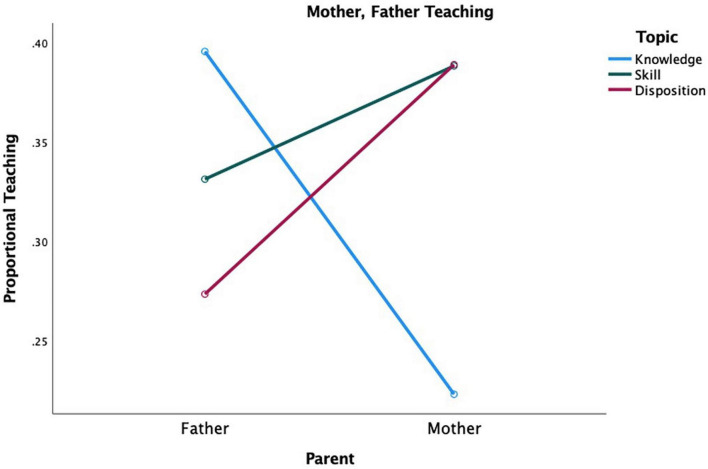
Mother, father teaching of knowledge, skills, and dispositions.

A *post-hoc* analysis indicated fathers (*M* = 0.47, *SE* = 0.05) and mothers (*M* = 0.48, *SE* = 0.05) were equally likely to teach their children, *F*(1,36) = 0.008, *p* = 0.93, $\eta_{\text{p}}^{2}$ = 0.00. Results comparing parents’ (mother, father) teaching of the domains of learning (knowledge, skills, dispositions) revealed an interaction, *F*(2,30) = 6.69, *p* = 0.004, $\eta_{\text{p}}^{2}$ = 0.31. Fathers (*M* = 0.40, *SE* = 0.05) were more likely to teach knowledge than mothers (*M* = 0.22, *SE* = 0.04), whereas mothers (*M* = 0.39, *SE* = 0.04) were more likely to teach dispositions than fathers (*M* = 0.27, *SE* = 0.05). This finding partially supports the hypothesis regarding parental differences in teaching knowledge and dispositions; however, contrary to expectations, mothers and fathers were equally likely to teach skills (*M* = 0.39, *SE* = 0.04; *M* = 0.33, *SE* = 0.04, respectively) (see Figure 1).

#### Parent Teaching by Sibling Birth Order and Gender

A comparison between parent teacher (mother, father) by learner (older or younger child) isolating dyadic teaching sequences showed a 2-way trend, *F*(1,29) = 3.83, *p* = 0.06, $\eta_{\text{p}}^{2}$ = 0.12. Counter to the expectation that both parents would be more likely to teach younger than older siblings, mothers were more likely to teach older (*M* = 0.54, *SE* = 0.05) than younger siblings (*M* = 0.46, *SE* = 0.05), whereas fathers were more likely to teach younger (*M* = 0.60, *SE* = 0.05) than older siblings (*M* = 0.41, *SE* = 0.05).

A series of one-way ANOVAs of parental teaching of domains of learning (knowledge, skills, dispositions) by child gender (girl, boy) separately by birth order (older, younger) indicated mothers were more likely to teach skills to their older daughters (*M* = 0.47, *SE* = 0.05) than older sons (*M* = 0.30, *SE* = 0.04), *F*(1,34) = 7.26, *p* = 0.01, $\eta_{\text{p}}^{2}$ = 0.18, supporting the hypothesis for mothers, but not for fathers. No differences were evident between mothers’ and fathers’ teaching of knowledge and dispositions to their older sibling child. Further, no differences were evident for parents’ teaching of domains of learning by younger sibling gender.

Next, *post hoc* one-way ANOVAs comparing fathers’ and mothers’ teaching of subtopics by gender revealed a trend in that fathers were more likely to teach prosocial dispositional behavior to their older sons than older daughters, *F*(1,31) = 3.77, *p* = 0.06, $\eta_{\text{p}}^{2}$ = 0.11. Whereas mothers were more likely to teach academic skills to their older daughters than older sons, *F*(1,34) = 3.56, *p* = 0.04, $\eta_{\text{p}}^{2}$ = 0.12. With respect to younger siblings, fathers taught more prosocial dispositional behavior to their younger sons than younger daughters, *F*(1,31) = 5.72, *p* = 0.02, $\eta_{\text{p}}^{2}$ = 0.16; mothers favored explaining problems, *F*(1,34) = 3.40, *p* = 0.07, $\eta_{\text{p}}^{2}$ = 0.09 (trend), and teaching cognitive skills, *F*(1,34) = 2.95, *p* = 0.09, η^2^ = 0.08 (trend), to their younger daughters than younger sons (see Table 4).

**TABLE 4:** Subtopics taught by child gender.

		Mother				Father			
		Older		Younger		Older		Younger	
		Female	Male	Female	Male	Female	Male	Female	Male
Domain	Sub-topic	*M* (*SE*)	*M* (*SE*)	*M* (*SE*)	*M* (*SE*)	*M* (*SE*)	*M* (*SE*)	*M* (*SE*)	*M* (*SE*)
Knowledge	Conceptual	0.07 (0.02)	0.08 (0.03)	0.07 (0.02)	0.08 (0.03)	0.11 (0.04)	0.05 (0.07)	0.07 (0.02)	0.09 (0.03)
	Academic	0.06 (0.01)	0.02 (0.01)	0.04 (0.01)	0.04 (0.01)	0.07 (0.03)	0.03 (0.02)	0.04 (0.02)	0.06 (0.03)
	Game rule	0.07 (0.03)	0.15 (0.05)	0.12 (0.04)	0.10 (0.04)	0.19 (0.25)	0.33 (0.07)	0.22 (0.06)	0.30 (0.07)
	Explanation	0.03 (0.02)	0.02 (0.01)	0.04 (0.02)^a^	0.01 (0.01)^b^	0.02 (0.01)	0.03 (0.02)	0.04 (0.02)	0.01 (0.01)
Skills	Cognitive	0.29 (0.06)	0.22 (0.03)	0.31 (0.05)^a^	0.20 (0.04)^b^	0.19 (0.05)	0.23 (0.06)	0.26 (0.06)	0.16 (0.04)
	Problem solving	0.15 (0.06)	0.07 (0.02)	0.08 (0.03)	0.14 (0.06)	0.03 (0.02)	0.05 (0.02)	0.02 (0.01)	0.06 (0.03)
	Academic	0.08 (0.03)^a^	0.02 (0.01)^b^	0.06 (0.02)	0.03 (0.02)	0.11 (0.06)	0.07 (0.03)	0.06 (0.03)	0.12 (0.06)
	Motor	0.01 (0.01)	0.02 (0.01)	0.02 (0.01)	0.00 (0.00)	0.02 (0.01)	0.03 (0.02)	0.03 (0.02)	0.02 (0.01)
Dispositions	Prosocial	0.12 (0.04)	0.08 (0.03)	0.09 (0.03)	0.10 (0.04)	0.03 (0.01)^a^	0.10 (0.03)^b^	0.02 (0.01)^a^	0.06 (0.03)^b^
	Social rules	0.19 (0.05)	0.32 (0.07)	0.23 (0.06)	0.29 (0.06)	0.26 (0.08)	0.15 (0.04)	0.26 (0.08)	0.14 (0.04)
	Self-control	0.05 (0.02)	0.08 (0.02)	0.05 (0.01)	0.09 (0.03)	0.04 (0.02)	0.03 (0.01)	0.04 (0.02)	0.03 (0.01)

*Superscript letters represent significant differences in proportional parent teaching of sub-topics between sibling gender (“a” is significantly different than “b” in each row).*

### Domains of Learning by Context

A context (contingent activity, conflict, game) by domains of learning (knowledge, skills, dispositions) analysis indicated an interaction, *F*(4,15) = 7.77, *p* < 0.001, $\eta_{\text{p}}^{2}$ = 0.67, partially supporting the hypothesis that during conflict, skills (*M* = 0.41, *SE* = 0.05) and dispositions (*M* = 0.57, *SE* = 0.06) were taught more than knowledge (*M* = 0.18, *SE* = 0.05). In partial support of the predictions, in a game context, knowledge (*M* = 0.55, *SE* = 0.06) was taught more than dispositions (*M* = 0.18, *SE* = 0.05), and no differences were evident with skills (*M* = 0.33, *SE* = 0.06). Contrary to the hypothesis, during contingent activity, there were no significant differences between teaching knowledge (*M* = 0.29, *SE* = 0.05), skills (*M* = 0.44, *SE* = 0.06) or dispositions (*M* = 0.32, *SE* = 0.05).

### Domains of Learning by Teaching Strategies

A 6 (strategy) by 3 (domain of learning) ANOVA indicated an interaction, *F*(5.14, 169.60) = 17.59, *p* = 0.000, $\eta_{\text{p}}^{2}$ = 0.35. Results partially support our hypothesis that skills would be taught through demonstration and dispositions with the strategies of direct instruction, positive, and negative feedback. Direct instruction was more likely to be utilized when teaching dispositions (*M* = 0.64, *SE* = 0.04) followed by knowledge (*M* = 0.38, *SE* = 0.05) and was least employed when teaching skills (*M* = 0.25, *SE* = 0.03). Labeling was more likely to be used when teaching knowledge (*M* = 0.49, *SE* = 0.05) than skills (*M* = 0.27, *SE* = 0.03) or dispositions (*M* = 0.24, *SE* = 0.04). Demonstration was more likely to be used when teaching knowledge (*M* = 0.15, *SE* = 0.03) and skills (*M* = 0.11, *SE* = 0.03) than dispositions (*M* = 0.02, *SE* = 0.01). Positive feedback was more likely to be used when teaching knowledge (*M* = 0.05, *SE* = 0.02) and skills (*M* = 0.04, *SE* = 0.01) than dispositions (*M* = 0.01, *SE* = 0.00). Negative feedback was more likely to be used when teaching dispositions (*M* = 0.34, *SE* = 0.05), followed by knowledge (*M* = 0.24, *SE* = 0.04), than skills (*M* = 0.14, *SE* = 0.03). Metacognitive strategies were more likely to be used when teaching skills (*M* = 0.71, *SE* = 0.06) than knowledge (*M* = 0.35, *SE* = 0.04) or dispositions (*M* = 0.39, *SE* = 0.04).

### Domains of Learning by Learner Response

An ANOVA for learner response (active involvement, compliance, no response) during the teaching of learning domains (knowledge, skills, dispositions) revealed a significant interaction *F*(4,26) = 2.68, *p* = 0.05, $\eta_{\text{p}}^{2}$ = 0.29. When skills were taught, children were more likely to be actively involved (*M* = 0.48, *SE* = 0.06) than comply (*M* = 0.27, *SE* = 0.04), but no difference in relation to not responding (*M* = 0.26, *SE* = 0.05). No differences existed between active involvement, compliance and no response when teaching knowledge (*M* = 0.37, *SE* = 0.05; *M* = 0.37, *SE* = 0.04; *M* = 0.25, *SE* = 0.04, respectively) or dispositions (*M* = 0.33, *SE* = 0.04; *M* = 0.39, *SE* = 0.04; *M* = 0.28, *SE* = 0.04, respectively). These findings did not line up with expectations that active involvement and no response would be the most common responses to the three topics taught.

## Discussion

Children absorb knowledge, develop skills, and build dispositional attitudes in their engagement in all social realms. Socioculturally, the home environment is considered the crux of children’s learning and development in the early years (Bornstein, 2015). This novel investigation of naturalistic parental teaching focusing on domains of learning and subtopics accounting for birth order, gender, context, teaching strategies, and learner response, provides valuable knowledge regarding the richness of learning between family members in a non-digitized home environment. Theoretically, findings highlight the intricacies and complexities of Vygotsky’s (1978) sociocultural notions in practice. This study serves as a platform from which empirical research can build knowledge on the teaching/learning process within different environments (e.g., digitized play, outdoor environments, leisure activities).

### Domains of Learning and Subtopics

Families are cultural hubs that nurture literacy skills, fostering different *functions* of literacy for communication and meaning making purposes (Wasik and Van Horn, 2012). As such, literacy represents cultural values and traditions *within* families; some families may focus on language skills and foundational literacy (i.e., print material and mathematics), whereas other families may follow an oral language approach. Based on the dominant empirical literature on numeracy and print literacy (e.g., Hood et al., 2008; Vandermaas-Peeler et al., 2012), we expected families from this sample population (i.e., white urban middle class) would teach knowledge and skills more than dispositions. Counter to expectations, and in support of Bloom et al. (1956) and Katz’s (1986), parents attended equally to all three domains of learning.

Comparing subtopics within each domain of learning, findings show certain types of knowledge, skills, and dispositions dominated the teaching/learning space. Specifically, game rules (knowledge), cognitive (skills), social and household rules (dispositions) were taught most often in comparison to other types of knowledge (i.e., academic, conceptual, explanation of a problem), skills (i.e., academic, problem solving, fine and gross motor) and dispositions (i.e., prosocial and moral, self-control). No hypothesis was posited for subtopics, thus this finding fills a gap in knowledge as to *what* is being taught in parent–children teaching episodes at home.

Studying subtopics provides a window into parents’ social and cultural values transmitted to children through teaching practices. Cognitive skills involved hypothesis testing (e.g., parent suggests children try to see if the bear bounces of the wall), testing knowledge (“When I twirl you, how do I do it? If I hold your hand what does it do to your wrist?”), deep thinking (“I know cheaters might win, but they never prosper”), perspective taking (parent explains how one might think or feel), and consequences of an action (e.g., parent tells their child that they are going to flip off the chair if they keep bouncing). Game rules (knowledge) involved general rules (parent explains the outcome of the game is a tie) or specific game rules (“You’re not supposed to know where the balloons are hidden”). Rule-based games included competitive board games (e.g., Snakes and Ladders), card games (e.g., go fish), or physical games (e.g., hockey). Social and household rules (dispositions) included common social courtesy (“Say thank you”), turn taking or sharing (“It’s Samantha’s turn to put this away”), or socially appropriate behavior (“Screaming is not necessary”). Examples show parents challenge children’s deep thinking, encourage independent thought, and foster connections between actions and consequences, game play and adherence to game rules, and social and household rules.

Teaching these topics reflects individualistic cultural values where social opportunities support autonomous thought and behavior, encourage competitiveness, and enforce social and household rules that pertain to personal responsibility (e.g., apologizing), dividing resources (e.g., each child can have one marker each), or providing limits to individual behavior (e.g., no running in the house) (Wainryb and Recchia, 2014). Materials used during teaching sequences included paper and pencils, scissors and glue, books, cards, game boards, dolls, trains, puzzles, hockey sticks, marbles, and stuffed animals. Evidently the interconnection between parents’ and children’s minds and selective cultural activities transfers social and cultural meaning and cultural tool knowledge (material and psychological) through the teaching/learning process (Fuhrer and Josephs, 1998).

Pertinent to the theoretical basis of the study, most teaching sequences (93%) involved two subtopics, while the remaining involved three subtopics. Thus, no teaching sequences focused on one topic in isolation, supporting the notion that guided participation hones sense making through the integrated nature of social-cultural experience (e.g., game rule and prosocial; cognitive skills and social rule; game rule and early academic skills). This also speaks to the value of children’s learning experiences in the home setting, in that families provide ongoing experiences through which children develop an understanding of their social and cultural world (Bornstein, 2015; Hedges, 2021).

### Father and Mother Teaching

This research studied sequences of teaching in which both parents were present, comparing mothers’ and fathers’ independent teaching episodes. Contrary to findings by Clarke-Stewart (1978) showing mothers’ interaction with their children (talk, responsiveness, and play) were reduced when fathers were present, our study indicates fathers and mothers were equally likely to teach their children when both were present.

In addition, teaching sequences were identified in the coding process where parents co-taught. Tallies indicate varied mother-father teaching dynamics; parents most often cooperated (34%), were equally likely to dominate (father = 16%; mother = 14%), and provided support (fathers support mothers = 11%; mothers support fathers = 25%). Though not assessed statistically, tallies illustrate the complexity of parent teaching, where children may be learning beyond explicit teachings (i.e., situational context), in which social experience itself translates meaning (Vygotsky, 1978). If parents cooperate in their teaching, children may learn how to cooperate or understand this is the norm in adult partnerships. If one parent dominates, this may reflect a power imbalance in parenting (Della Porta et al., 2021). In this case, children may learn this style is representative of adult relationships and if a child identifies with that parent, they may engage in dominating behavior themselves.

#### Domains of Learning and Subtopics

Regarding the three domains of learning, fathers were more likely to teach knowledge, whereas mothers were more likely to teach dispositions. In line with literature (Power and Parke, 1986; McBride and Mills, 1993; Pleck, 1997; Paquette and Bigras, 2010), mothers are more involved in discipline and enforcing social and household rules, whereas fathers are more involved in translating knowledge through playful activities. Counter to Paquette (2004) and Pleck (1997) who reported fathers reinforce and teach skills, our study showed mothers and fathers were equally likely to teach skills. This discrepancy in findings is likely due to the means through which skills are studied. For instance, in Paquette’s work, skills refer to competitive (e.g., physical fighting, assertiveness, quick decision making) and conflict resolutions skills. According to Paquette, these skills are core to industrialized societies and individualistic cultures. Whereas our study covers a breadth of skills from academic to gross and fine motor. Thus, it appears parents teach a wide range of skills or cultural tools.

#### Birth Order and Gender

The Vygotskian approach endorses teaching according to competency (Pellegrini et al., 1985), hence it was predicted parents would teach younger siblings more than older. However, findings revealed differences based on parent gender; mothers were more likely to teach older siblings, whereas fathers were more likely to teach younger siblings. It can be argued that the difference is due to the age/stage of the child’s development. Arguably, insight into a child’s behavior and play cannot be considered outside of the sociocultural context (Edwards, 2014). To elaborate, 4-year-old younger siblings, at the preschool stage, are developing self-confidence and typically require adult attention and approval. Children at this age are very physical in their activities (e.g., gross motor, arts, taking physical risks) (Hartley and Goldenson, 1970). Whereas for 6-year-olds, entering school-age, become refined in motor and intellectual skills developing a sense of industry, and becoming more independent (Delvecchio et al., 2016). Due to these developmental changes, older children tend to be attracted to games with rules (Piaget, 1962). Thus, mothers may gravitate toward challenging older siblings intellectually, with academic and learning-related activities, whereas fathers may engage responsively more with younger siblings in physical play (Schoppe-Sullivan et al., 2013; Foster et al., 2016).

As for domains of learning, as predicted, mothers taught skills to their older daughters more than older sons. Counter to expectations, fathers were equally likely to teach skills to their older daughters and sons, instead of our prediction (i.e., fathers would teach skills more to sons than daughters). Further investigating these differences by comparing subtopics, findings indicated mothers taught academic skills more to their older daughters than sons and explained problems (knowledge) and cognitive (skills) to their younger daughters than sons; fathers taught more prosocial dispositional behavior to both older and younger sons than daughters.

Looking at academic skills and a higher cognitive demand, the mother-older daughter finding is in line with Maccoby and Jacklin (1974), who proposed that parent interactions with children based on gender may be influenced by birth order constellation. Specifically, mothers may be more severe with first-born daughters than second-born daughters and vice versa for fathers. In direct contrast, Bell et al. (1981) found that the same-sex severity and opposite sex indulgence rule applied more strongly to fathers than mothers, particularly in three-child families. Relatedly, and counter to our findings, McGillicuddy-De Lisi (1988) discovered parents demand high-level cognitive skills from children of the opposite sex. We found fathers were more likely to teach sons about prosocial disposition, whether older or younger; this finding was contrary to research showing both parents demand high-level cognitive skills and counter to the same-sex severity and opposite sex indulgence birth order constellation proposal. Thus, this study provides insight into *what* areas mothers and fathers focus on in teaching their children. Bell et al. (1981) argue when conducting research on families, findings may be “artificial” (p. 704) unless family constellation is taken into account. This is recommended for future research, among other consideration of family dynamics, including power dynamics, as the study of the family is a complex endeavor (Della Porta et al., 2021).

Regarding mothers’ explanation of problems (knowledge) and teaching cognitive (skills) more to younger daughters than sons, the same argument by Maccoby and Jacklin (1974) may apply (without considering birth order constellation). That is, mothers may either demand more cognitive sophistication from daughters or believe daughters need more support in that domain (Frome and Eccles, 1998). Particularly with younger daughters, mothers may perceive their ZPD requires more attention for appropriate cognitive challenge (Vygotsky, 1978; Rogoff, 1990). If this is the case, it appears mothers are more demanding of daughters in the cognitive developmental realm, and are responsive to daughters’ level of intellect, responding to their needs through differing social engagement, an appropriate meeting of minds (Fuhrer and Josephs, 1998).

#### Context

In partial support of Farhat et al.’s (2021) findings, skills and dispositions were taught more during conflict, whereas knowledge was taught more during games. Contrary to the hypothesis, during game play, skills was not a salient teaching domain; during contingent activity, there were no significant differences between the teaching of knowledge, skills, or dispositions. During conflict, it seems altercations surround more explicit differences in mental processes or physical procedures (i.e., skills) and socially and morally appropriate behavior (i.e., dispositions). Notably, cognitive (skills) co-occurred with disposition categories of prosocial behavior (*n* = 12) and social and household rules (*n* = 10). For instance, when developing a social and moral conscience, parents apparently teach perspective taking or facilitate children’s understanding of the implications of their actions, which are evidently elicited in conflict (Turiel, 2014). The cognitive (skills) and prosocial and moral combination (dispositions) tended to occur in situations where parents ask the child to rethink their behavior and perspective take (e.g., “That’s not a very nice thing to do though, is it? How would you like it if somebody did it to you?”; “Now, don’t you go and take things. You do to Samantha what you want her to do to you”). The cognitive (skills) and social/household rules (dispositions) combination typically occurred when parents reminded children about known social or household rules or explained the connection between a social/household rule and the consequence of their action (e.g., “Listen, those aren’t for playing with. Those are to go on a Christmas present. If you break them, they won’t get on the tree”; “You know better, just because he does something doesn’t mean you do it too”). This further outlines the complexities of sociocultural interactions, in that social interactions involve multiple mental considerations relating to culturally appropriate behavior (Vygotsky, 1978).

In the game context, it was surprising that knowledge not skills was taught most often. As indicated above, this context typically involved games with rules, including table games, such as cards or board games, or floor games, such as hockey or marbles. Based on materials available and offered to children in the home environment, knowledge was taught in the playing of the games, as they require adherence to pre-determined rules. Thus, it is likely that skills were not as readily teachable during such games. This finding shows the impact of cultural tools on children’s learning, in this case, about conforming to the rules laid out by the game (Stone, 2004). Arguably, if the activities offered were social in nature with a shared goal, the domain of learning would relatedly differ, for instance, skills would engage learners in a collaborative activity, scaffolded by the adult (Warneken et al., 2012).

#### Teaching Strategies

Socioculturally speaking, these findings show *how* parents teach their children across domains of learning. In line with expectations, skills were strengthened through practice (i.e., demonstration) (Katz, 1986) and metacognitive strategies were used to teach skills. This provides evidence of the joint mental attention required for transmission of intellectual skills and flexibility (e.g., questioning, triggering thought processes, suggesting actions to consider) (Fuhrer and Josephs, 1998). As for dispositions, in line with Katz (1986), parents taught using direct instruction and negative feedback, which seems appropriate in that social or household rules and prosocial and moral expectations are dictated by parental authority and pre-set societal and cultural norms (Turiel, 2014). Contrary to expectations, dispositions were not encouraged through reinforcement. Breaking these rules may elicit negative emotions for parents such as feeling disrespected, and therefore may be frowned upon when children act upon impulses eliciting direct instructions (e.g., “stop,” “don’t do that,” “that’s enough”) and/or negative feedback (e.g., “it’s not your turn,” “not like that,” “that’s no right”). In terms of knowledge, no predictions were made; findings indicate that parents used labeling, demonstration, and positive feedback to teach knowledge. Based on the means through which domains of learning are taught, it seems knowledge is more positively valued and encouraged in these families. Further research is warranted to investigate differing ways domains of knowledge are taught and how this impacts children’s learning in these core areas of education.

#### Learner Response

In the family teaching literature (i.e., parent–child and sibling), children are more likely to be actively involved and not respond to teaching than comply (Howe et al., 2015, 2016; Farhat et al., 2021). We found, however, when skills were taught, children were more likely to be actively involved than comply. No differences existed between the three types of responses (active involvement, compliance, and no response) when parents taught knowledge or dispositions. As such, skills appear to actively engage children in the learning process. This is not to say that children do not learn from other domains – quite the opposite – we argue that children learn in many forms – but the most engaging for children is the active process of skill development. Furthermore, teaching skills most often involved cognitive skills (e.g., deep and critical thinking, perspective taking, consideration of consequences), as well as academic, problem solving, and fine and gross motor skills. These types of activities whether mental or physical appear to engage the child– entice questioning, commenting, or expanding the teaching.

It seems skill development is more likely to involve social engagement by both parties, whereas knowledge is transmitted from the more knowledgeable individual and dispositions (social and household rules) are something the adult as the expert imparts to the child (Vygotsky, 1978; Rogoff, 1990; Fuhrer and Josephs, 1998). In teaching these domains it appears there is an equal likelihood of a child actively or passively engaging, which is understandable since knowledge and dispositions are not often disputable.

## Conclusion

This study adds to the literature by identifying *what* parents are teaching their children and *how* the teaching process varies based on socially constructed behavior (e.g., gender norms and expectations) and situational context (e.g., actors involved) in the home setting. Though this study investigated explicit teaching, there is a wealth of learning that occurs implicitly. In the teacher-learner relationship, individuals interact in ways that eventually result in the acquisition of skills and knowledge by the learner and ultimately their successful completion of a task on their own (Vygotsky, 1978). Children observe occurrences in the home, for example adult activities, marital conflict, sibling interaction in families with three or more children, and cultural activities (e.g., cooking, event planning, family rituals). Often, it is a child’s active engagement that initiates explicit teaching, such as attention to the activity or questioning. Thus, future research on parent–child teaching should include an examination of the initiation of teaching in their assessment of the teaching/learning process.

In terms of statistical limitations, most analyses, particularly repeated measures ANOVAs have strong power ranging from 0.82 to 1.00, yet some significant one-way ANOVA findings were underpowered, thus readers should be cautious when interpreting these findings. Further, this sample consisting of white urban middle-class families represent typical individualistic cultural values, limiting generalizability of findings. In this cultural context, language and non-verbal communication were used to isolate the teaching/learning processes. Materials or cultural tools are just as important in the study of sociocultural interaction; hence, it is recommended that cultural context and related tools (psychological and material) be included in future research on parent–child teaching. Relatedly, findings are based on two-parent family interactions in which both parents were present, limiting generalizability to other family configurations. We encourage researchers to study families in all forms to best understand family functioning in an inclusive manner. We also suggest investigating parent teaching with only one parent present to study how that behavior dynamic would change during the parent teaching or scaffolding compared to when two parents are present.

Finally, data collection began in 1989, a time in which technology was only beginning to make strides in the social-cultural landscape, nevertheless television watching was a major activity in the lives of most families. Therefore, this study sets somewhat of a baseline for future research investigating parent–child teaching in other informal learning contexts, beyond the non-digitized indoor setting (Rogoff et al., 2016), especially with continuing advances in our globalized and digital world. This can involve parent–child teaching using digital tools or interactions in outdoor settings, assessing culturally represented learning tools. Our cultural-historical context sets the stage for how we become accustomed to sociocultural ways of literacy development (communication and making meaning) (Wasik and Van Horn, 2012). Such investigations would provide further insight into parental facilitation and scaffolding of children’s learning and development. Whether it be natural, digital, or other sociocultural environments, the teaching will likely differ. This paper provides a first look at the topics taught in an informal learning environment within which young children are deeply connected.

## Data Availability Statement

The datasets presented in this article are not readily available because “Participants have not agreed that their data be shared.” Requests to access the datasets should be directed to NH.

## Ethics Statement

The studies involving human participants were reviewed and approved by HR, University of Waterloo, and NH, Concordia University. Written informed consent to participate in this study was provided by the parents/guardians for their own and their children’s participation.

## Author Contributions

SDP, NH, and FF contributed to conception and design of the study. SDP and PS co-developed the coding scheme and coded data, and wrote the “Introduction” section. HR contributed the data and original coding. SDP conducted the statistical analysis and wrote the “Results” and “Discussion” sections. PS wrote the “Materials and Methods” section. NH contributed the data and expertise in the field, as well as read and revised the manuscript. All authors contributed to, read, and approved the submitted version.

## Conflict of Interest

The authors declare that the research was conducted in the absence of any commercial or financial relationships that could be construed as a potential conflict of interest.

## Publisher’s Note

All claims expressed in this article are solely those of the authors and do not necessarily represent those of their affiliated organizations, or those of the publisher, the editors and the reviewers. Any product that may be evaluated in this article, or claim that may be made by its manufacturer, is not guaranteed or endorsed by the publisher.
